# Optical manipulation: advances for biophotonics in the 21st century

**DOI:** 10.1117/1.JBO.26.7.070602

**Published:** 2021-07-07

**Authors:** Stella Corsetti, Kishan Dholakia

**Affiliations:** a University of St Andrews, SUPA, School of Physics and Astronomy, St. Andrews, United Kingdom; b University of Adelaide, School of Biological Sciences, Adelaide, South Australia, Australia; c Yonsei University, College of Science, Department of Physics, Seoul, Republic of Korea

**Keywords:** optical manipulation, optical tweezers, trapping, optics, biophotonics, light

## Abstract

**Significance:** Optical trapping is a technique capable of applying minute forces that has been applied to studies spanning single molecules up to microorganisms.

**Aim:** The goal of this perspective is to highlight some of the main advances in the last decade in this field that are pertinent for a biomedical audience.

**Approach:** First, the direct determination of forces in optical tweezers and the combination of optical and acoustic traps, which allows studies across different length scales, are discussed. Then, a review of the progress made in the direct trapping of both single-molecules, and even single-viruses, and single cells with optical forces is outlined. Lastly, future directions for this methodology in biophotonics are discussed.

**Results:** In the 21st century, optical manipulation has expanded its unique capabilities, enabling not only a more detailed study of single molecules and single cells but also of more complex living systems, giving us further insights into important biological activities.

**Conclusions:** Optical forces have played a large role in the biomedical landscape leading to exceptional new biological breakthroughs. The continuous advances in the world of optical trapping will certainly lead to further exploitation, including exciting *in-vivo* experiments.

## 1 Introduction

Half of the Nobel Prize in Physics in 2018 was awarded to Arthur Ashkin for the groundbreaking invention of optical tweezers. The committee particularly recognized the impact that optical tweezers (and optical forces more generally) have had on biology. Ashkin’s first demonstration of optical forces was presented in 1970.^1^ In that study, inert particles were held by two opposing, gently focused light beams, the counterpropagating dual-beam trap. The major advance occurred 16 years later with the advent of optical tweezers.^2^ In this embodiment, a single tightly focused laser beam exerted a sufficient force to hold a microscopic particle very close to the focal point of the laser field.

Optical forces have made a seminal impact for manipulating objects from the size of an individual atom right up to a large cell or embryo.^3^^,^^4^ The breadth of applications is remarkable. From a physics perspective, the optical trapping has impacted our understanding of the very nature of linear and angular momentum of light, been used for various studies in microfluidics, allowed us to explore nonequilibrium thermodynamics as well as contributing to the field of levitated optomechanics. This is aiming toward a deeper understanding of the transition between classical and quantum physics, as well as developing exquisite high precision sensors.

How can light exert a force? Light possesses momentum and the propagation of light between various media results in a change in its propagation, which is a transfer of momentum and therefore results in a force. This interaction may be described in a number of ways depending on the size of the object versus the trapping wavelength used. The optical forces used to confine particles of interest that are much larger than the wavelength of the light (the Mie regime) can be considered in this ray-optics picture, while particles much smaller than the trapping wavelength (the Rayleigh regime) are modeled as individual electric dipoles, which are drawn to high or low intensities due to the interaction between this dipole and the oscillating electric field of the light.^5^^,^^6^ The advantage of the optical traps is not only in exerting a force but also the fact that this can be readily measured. An optical tweezers is calibratable and acts as a Hookean spring. As a result, the force is directly proportional to the displacement.

In this perspective, we do not aim to cover all the progress in trapping but rather discuss some of the key advances this century, with emphasis on the last 10 years, focusing on those of importance for biophotonics. This period has seen optical tweezers consolidate and expand their capabilities to a large extent. In particular, they have enabled the study of more complex living systems and deepened our understanding of fundamental biological processes. In Sec. 2, we will discuss how the direct determination of forces in optical tweezers on arbitrarily shaped objects, and in multiple traps simultaneously, has emerged as an important theme. We then progress to discuss multimodality: traps have traditionally been combined with other optical techniques. As a recent advance, we will describe the recent unified use of optical and acoustic traps that offers advantages including studies across different length scales. We then highlight the progress made for confinement at the very small scale, in direct trapping of single-molecules and even single-viruses with optical forces (Sec. 3). In Sec. 4, we detail the use of optical tweezers to trap single cells focusing on the possibility to manipulate them *in vivo*, including in living animals. Finally, we conclude by suggesting possible future directions for the use of optical trapping in biophotonics.

## 2 Advances for Quantitative Force Measurements in Biological Systems

Historically, one of the main advantages and uses of optical tweezers in biophysics is as a tool for measuring both forces and displacement. The precise knowledge of forces provides insight into molecular dynamics, a topic that is expanded upon in Sec. 3. This includes DNA dynamics and the motion of molecular motors, crucial for many intercellular processes. It also allows us to explore the mechanical properties of cells, providing us with information about their elastic and viscoelastic properties. Traditionally, precision measurement in optical tweezers has been undertaken using various methods. As the optical trap is a spring, determining the spring constant (trap stiffness) plays a central role for precise measurement. This has been achieved using methods of hydrodynamic drag, use of the equipartition theorem or the use of back focal plane interferometry (BFPI) using a quadrant photodiode (QPD). For a more in-depth discussion of optical tweezers and how they measure forces and displacement, we refer the reader elsewhere.^7^^–^^10^

Fundamentally, one may consider the exerted force is equal to the change of the momentum flux of the trapping beam. Therefore, recording the momentum of the scattered light directly from the particle is itself a measure of the optical force. This consideration leads to the concept of a direct force measurement in contrast to previous approaches and has been a topic of recent interest in the field. This requires us to collect all of the scattered light from the particle. A positive attribute is that such an approach obviates any detailed information of physical parameters [e.g., size, shape, or refractive index (RI)] of the trapped specimen. This is thus particularly amenable to measurements inside biological organisms. To date, such direct force measurements^11^^–^^14^ recorded only the radial component of optical forces, whereas ideally the axial force should also be determined. In recent work, Thalhammer et al.^15^ recorded not only the far-field light distribution in the forward direction but also the total amount of backward scattered light to obtain accurate values of the axial force.

**Fig. 1:**
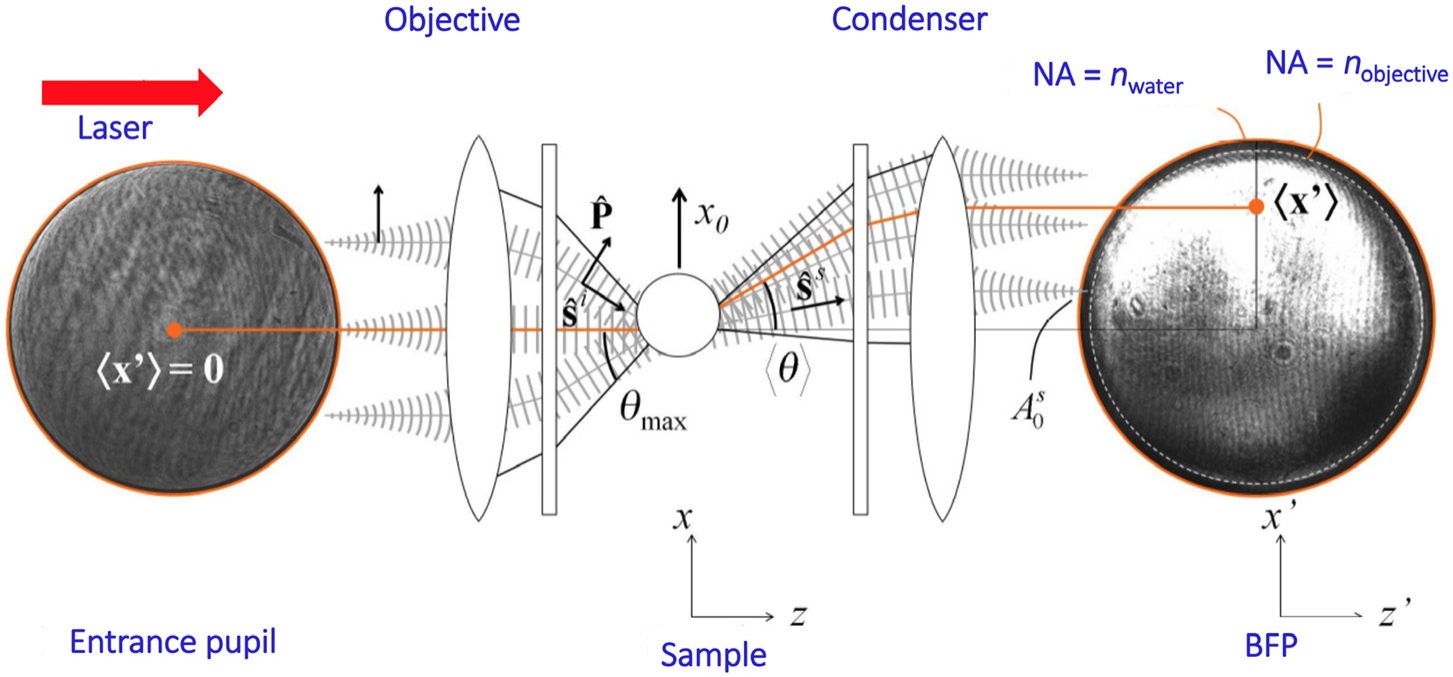
Schematic showing the principle of BFPI. The diagram shows the initial structure of the beam at the front focal plane of the objective and the changes due to the presence of the sample. The focused laser beam is scattered in all directions after interacting with the sample. The light pattern at the BFP of the condenser lens is symmetric, as it is for the incident beam, and the detector signal is zero when the sample remains at the center of the trap. When the position of the trapped sample relative to the incident beam varies, the distribution of light at the BFP changes. Reproduced with permission from Fig. 4 of Ref. 13, © 2012 Optical Society of America (OSA).

The direct measurement of forces exerted by an optical trap is in fact closely related to the use of BFPI. This measures displacements of optically trapped objects with a very high spatial and temporal resolution.^13^ Biological samples typically have a small RI mismatch with their surroundings and therefore most of the light (>95%) is scattered in the forward direction. This light can be collected by a high numerical aperture lens, and the measurement of the optical force may be achieved by a photodetector placed in a plane conjugate to the back focal plane of the condenser. A measure of the light momentum can be obtained using a position-sensitive detector (PSD). This measures the center of mass of the light distribution more accurately than a QPD which is commonly employed in BFPI.^15^ The principle behind BFPI is shown in Fig. 1.

The measured beam position and beam power are then used to estimate the radial components of the momentum and thus, the radial components of optical forces in traps. However, to go beyond the radial direction and gain insight into the sample’s behavior along the beam axis is a challenge.

**Fig. 2:**
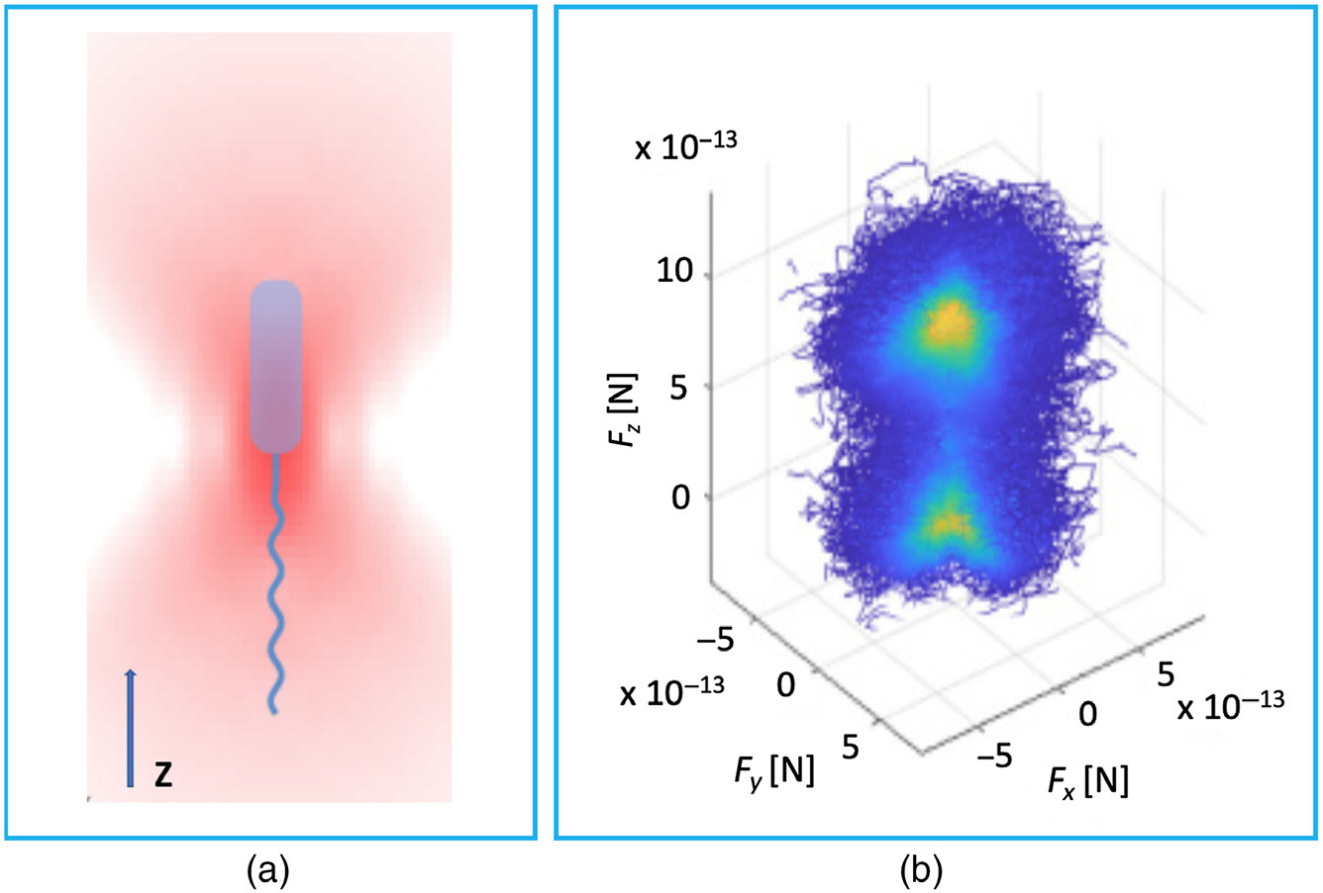
The force distribution in three dimensions for trapped *E. coli*. (a) Schematic of an *E. coli* held in Gaussian beam optical tweezers. (b) 3D representation of force contributions ($F_{y}$ and $F_{x}$ represent forces in the focal plane and $F_{z}$ is the axial contribution) for an optically trapped motile *E. coli*. Adapted with the permission from Figs. 3(a) and 4(d) of Ref. 16, © 2020 OSA.

To acquire both radial and axial force measurements, the direct force measurement method needs to be combined with other strategies.^15^^,^^16^ Armstrong et al.^16^ combined the use of a PSD with the use of position-sensitive masked detection (PSMD), selectively reflecting light in different directions using an appropriately defined mask (details about the mask can be found in Ref. 17), to perform full three-dimensional (3D) direct optical force measurements of trapped single *Escherichia coli* (*E. coli*) cells. Figures 2(a) and 2(b) show the schematic of a trapped motile *E. coli* and the 3D representation of optical force contributions, respectively. The 3D force is measured with both the PSD (position of the beam and radial forces) and PSMD (spread of the beam and axial force). They imaged the back focal plane of a condenser collecting the intensity distribution of the light scattered by the cells on the force detectors and used a CMOS camera to track the position of the cells. Using such a method, they were capable of capturing the motility properties of the microorganisms determining their swimming force and behavior. They obtained an average propulsive force of ∼0.53  pN carrying out 3D force measurements on individual cells. This value is very similar to the 0.5-pN force obtained from the measurements on wild-type *E. coli* confined to a microfluidic channel carried out by Chattopadhyay et al.^18^ The correlation of the direct force measurements in 3D with the position data acquired with the high-speed camera enabled them to distinguish between swimming and tumbling modes of microorganisms. This opens up the possibility to probe arbitrary-shaped biological swimmers regardless of their orientation in the trap.

**Fig. 3:**
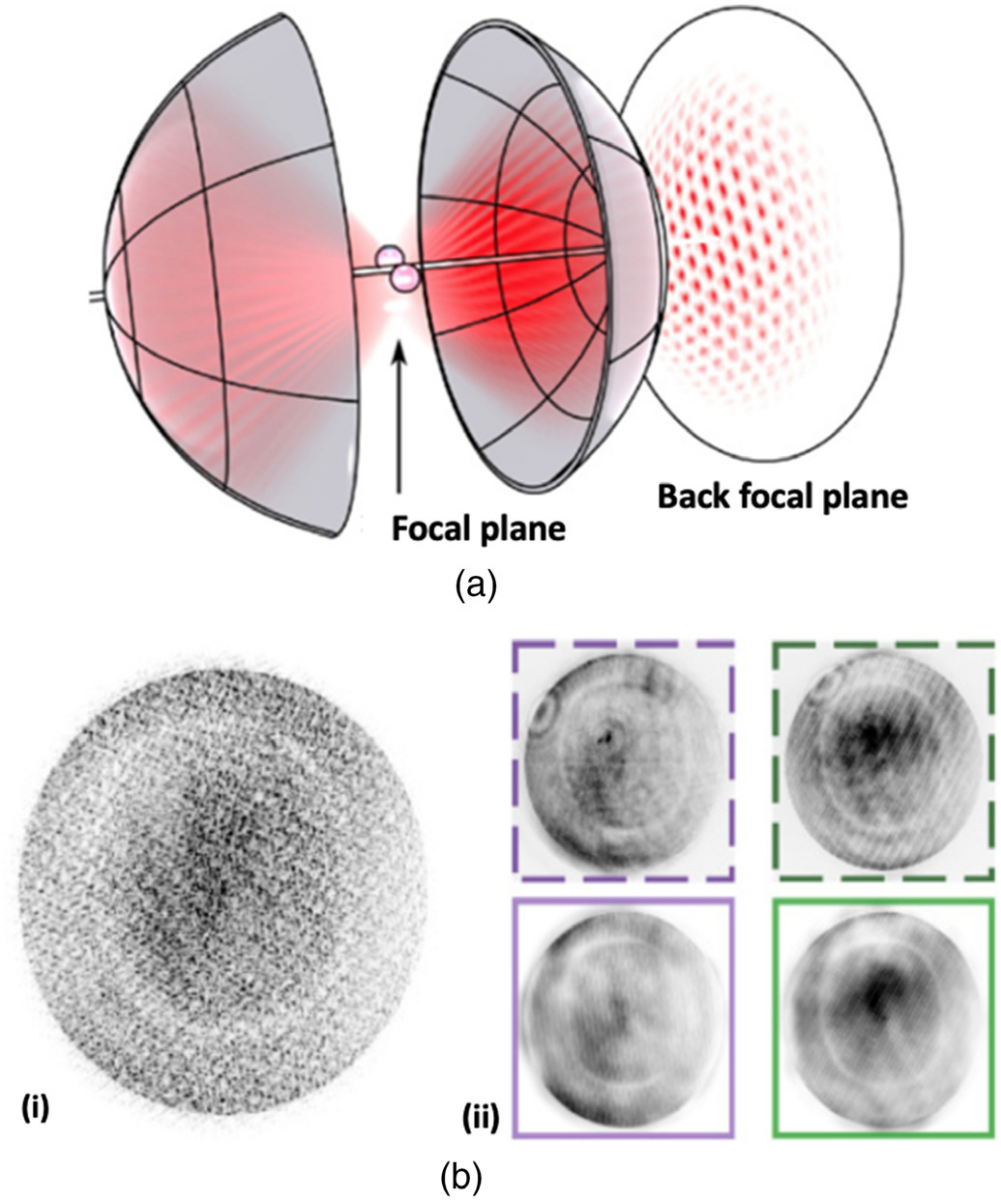
Far-field method allowing the simultaneous measurement of forces on multiple optically trapped particles. (a) Schematic of the intensity pattern in the back focal plane created by two trapped microspheres. (b) (i) Observed far-field intensity pattern for simultaneous measurement with 10 microspheres. (ii) Single-trap far-field intensity pattern for two of the 10 traps. The comparison between measured (top) and reconstructed (bottom) intensity patterns is shown. Adapted with permission from Figs. 1 and 4 of Ref. 19 (CC BY 4.0).

Recently, the direct force measurement method has addressed the case of multiple trapped particles, where there is overlapping of light in the far-field (back focal plane of the condenser). The development of a new method based on holographic force measurements allowed the deduction of individual forces for several optical traps simultaneously by disentangling the contribution of individual scattered fields from each of the trapped particles.^19^ By detecting the single interference pattern occurring in the back focal plane, the team numerically reconstructed the full complex light field in the focal plane, where the light fields of individual scatterers are well separated, determining the forces individually. Figure 3(a) shows the schematic of the intensity pattern in the back focal plane created by two trapped particles. Figure 3(b) shows (i) the observed far-field intensity pattern for simultaneous measurement of 10 microspheres and (ii) the single-trap far-field intensity pattern for two of the 10 traps.

Overall, these direct force studies open up the possibility of performing quantitative force measurements in more complex biological settings such as cells in the bloodstream.

New numerical methods for force determination have also emerged. These hold promise for biomedical studies. The Volpe group demonstrated microscopic force reconstruction via maximum-likelihood-estimator analysis to recover the force field acting on a Brownian particle from the analysis of particle displacements.^20^ Close to the equilibrium position, the force field may be modeled as a linear function of distance. This approach added an inherent simplicity and speed to such studies and could be applied to both conservative and nonconservative force fields.

### 2.1 Trapping Across Scales I: Confining Smaller Particles

In the previous section, we discussed direct recording of optical forces in both radial and axial directions. In addition to an increase in the resolution and the complexity of the system that can be probed, a wider dynamic range of measurements and sample sizes may be desirable.

The forces of light in the form of optical tweezers and the counterpropagating dual beam optical trap can in fact hold biological samples spanning size scales from ∼ sub ∼1 to 100  μm.^5^ Optical tweezers and the dual beam trap are far-field systems and thus subject to the diffraction limit of light. This limits the prospect of creating very strong field gradient forces. Though standard optical tweezers can trap nanometer-sized particles of high polarizability (e.g., metal nanoparticles), reverting to the near-field can create stronger field gradients and has the ability to directly confine single molecules or other nanometer-sized biological specimens. This may be achieved with plasmonic traps or photonic crystal optical tweezers.^21^^,^^22^ These are discussed in more detail in Sec. 3.2.

### 2.2 Trapping Across Scales II: Confining Larger Particles

The counterpropagating dual beam trap has been versatile for confining larger particles and for cell stretching, due to its use of divergent beams. This has advanced in the last decade to macrotweezers: this uses reflection from a mirror to create traps capable of confining highly motile organisms, such as *Euglena* protists and dinoflagellates of up to 70  μm length, three-dimensionally in cubic millimeter volumes.^23^ To trap even larger objects, we may turn from optical forces to acoustic forces: these can handle specimen sizes from a few hundred micrometers to millimeters. Optical trapping is better suited to the precise manipulation of individual small particles, but the comparatively weak optical forces are a limiting factor in trapping large objects. On the contrary, acoustic forces enable the levitation of much larger particles with lower acoustic field intensities compared with what is possible with optical field intensities. This is an advantage in terms of impact on the viability of trapped cells or micro-organisms. However, if the particle size is smaller than the acoustic wavelength, the exerted force scales with the volume of the trapped particle making it difficult to handle single small particles with acoustic forces. Acoustic trapping can manipulate objects down to the nanoscale if porous metal nanoparticles of high compressibility are used.^24^

Acoustic and optical forces allow noncontact forces to be applied to a biological specimen. However, the optical and acoustic contrast parameters that dictate the trapping forces are fundamentally different. In optical trapping, the dielectric polarizability and the relative refractive indices of the particle and the medium are key contrast parameters. In acoustic trapping, the mass density and compressibility of the particle with respect to the host fluid determine the acoustic contrast. The small density differences between cells and water often give sufficient acoustic contrast for the manipulation of single cells or microorganisms. This allows acoustic tweezers to work analogously to optical tweezers in many biologically relevant studies. Furthermore, the different sources of contrast in optical and acoustic trapping increase the number of options for manipulation in any given scenario. For an in-depth discussion about the differences between light and sound and the principles behind their ability to trap objects at different length scale objects, we refer the reader elsewhere.^25^ Here, we instead focus on the main hybrid applications of these two trapping modalities. In fact, as discussed above, optical and acoustic trapping are mainly complementary in terms of size scales. Acoustic trapping is capable of levitating entire living organisms, such as a *Caenorhabditis* *elegans* nematode,^26^ whereas, the optical trapping better suited to manipulate smaller objects. Therefore, the combination of the two modalities allows for a simultaneous investigation at both the microscopic and mesoscopic scales. One possibility, for example, is to use acoustic trapping to confine a sample and optical trapping for fine manipulation of different parts of the sample.

The macrotweezers dual-beam trap developed by Thalhammer et al.^23^ is capable of trapping large organisms. This has proven to be a convenient geometry to combine with acoustic fields.^27^ This combination allowed the manipulation of actively swimming micro-organisms in a microfluidic environment (*Euglena gracilis*).

The study demonstrated the implementation of active particle sorting realizing a form of invisible, reconfigurable microfluidic device. They used acoustic forces to confine particles in different planes, between which particles were selectively pushed with optical forces.

The combination of acoustic and optical forces has also been used to determine the mechanical characteristics of single cells. Yang et al.^28^ trapped a cell in a chip and made use of optical stretching for optical deformability and acoustophoresis for acoustic compressibility.

Calibrated optical tweezers have also proven to be useful to map acoustic force fields.^29^^–^^31^ This is particularly relevant for the design of acoustofluidic devices that can be used to control micrometer-sized objects in medical, chemical, and biological applications. Recently, Lamprecht et al.^31^ used an optically trapped particle as a probe to measure acoustically generated forces in 3D. The optical trap was combined with an acoustofluidic device to measure acoustic forces exerted on particles. They were capable of achieving sub-pico Newton force resolution by measuring the displacements in all directions of the trapped probe-bead with decoupled QPDs.

The experiments discussed are examples that show the combination of optical and acoustic trapping. This has proven to be successful in extending the range of biological studies that can be performed and this trend is expected to continue.

## 3 Optical Tweezers at the Nanoscale: Single Molecules and Viruses

Some of the most prolific experiments with optical trapping have been the study of individual single-molecules. Optical tweezers provided a step change for this area offering a complementary and different perspective with respect to traditional ensemble-based molecular studies.^32^ Many aspects of molecular thermodynamics and kinetics are challenging to investigate in bulk experiments, and fascinating details can only be unveiled by studying individual molecules. Since their conception, optical tweezers have successfully employed inert trapped microparticles as handles to attach to and monitor molecular dynamics. Studies have included DNA, kinesin, and the actin-myosin system. This section summarizes some of the main results from single-molecule experiments performed with the optical tweezers in the last 10 years, highlighting the new insights gained using this technique.

Nanoscale objects, which include small proteins and single viruses, are challenging to trap in traditional optical tweezers. While the optical scattering force scales with the sixth power of the particle size and therefore becomes negligible compared with the optical gradient force for very small objects, the optical gradient force itself (and thus the trap depth) scales with the third power of the particle size for a dilectric particle, and with the intensity gradient of the light. Traditional free-space optical tweezers, therefore, require exceptionally high powers to stably confine nanoparticles, which may come at the cost of photodamage. Optical tweezers cannot readily manipulate nanoscopic objects and this prevents easily trapping of minuscule molecules such as DNA/RNA or proteins.

Recent advances in materials science,^22^ along with the advent of nanotechnology and molecular biology, allowed optical tweezers to reach unprecedented force- and spatial-resolutions, opening up the door for new single-molecule experiments. In particular, the development of near-field traps with an increased gradient field allows direct single protein manipulation at low optical powers and paves the way for the development of devices capable of detecting single viruses.^33^

### 3.1 Single Molecule Studies Enabled by Optical Tweezers

Optical tweezers typically measure forces up to the piconewton (pN) scale. Importantly, protein–protein interactions as well as the forces involved in the overstretching transition of the DNA molecule and the forces produced by many molecular motors fall within the 1 to 70 pN range, making optical tweezers suitable to be used in such investigations. Molecular motors are enzymes that convert chemical energy coming from the adenosine triphosphate into mechanical energy and are responsible for most forms of movement in cells. The main molecular motors are myosin and kinesin, but DNA and RNA-polymerase (RNApol) can also be considered as molecular motors. Since the advent of optical tweezers, the elastic properties and modulation of biological properties of DNA molecules have been extensively studied.^34^^,^^35^ In this regard, one of the major observations has been the structural transition, referred to as the DNA overstretching transition, occurring when torsionally unconstrained double-stranded (ds) DNA is pulled from its ends with a force >65  pN. After this transition, the DNA is stretched to about 1.7 times its initial length.^36^^,^^37^ Since its discovery in 1996, the mechanism of this transition and the nature of overstretched DNA have led to substantial controversy. In fact, the main question to resolve is if the structure of overstretched DNA consists of two separated strands of single-stranded DNA (ss-DNA) formed as a result of melting or is due to a unique elongated form of dsDNA (S-DNA) resulting from a hypothetical B-to-S transition.^36^^,^^37^

**Fig. 4:**
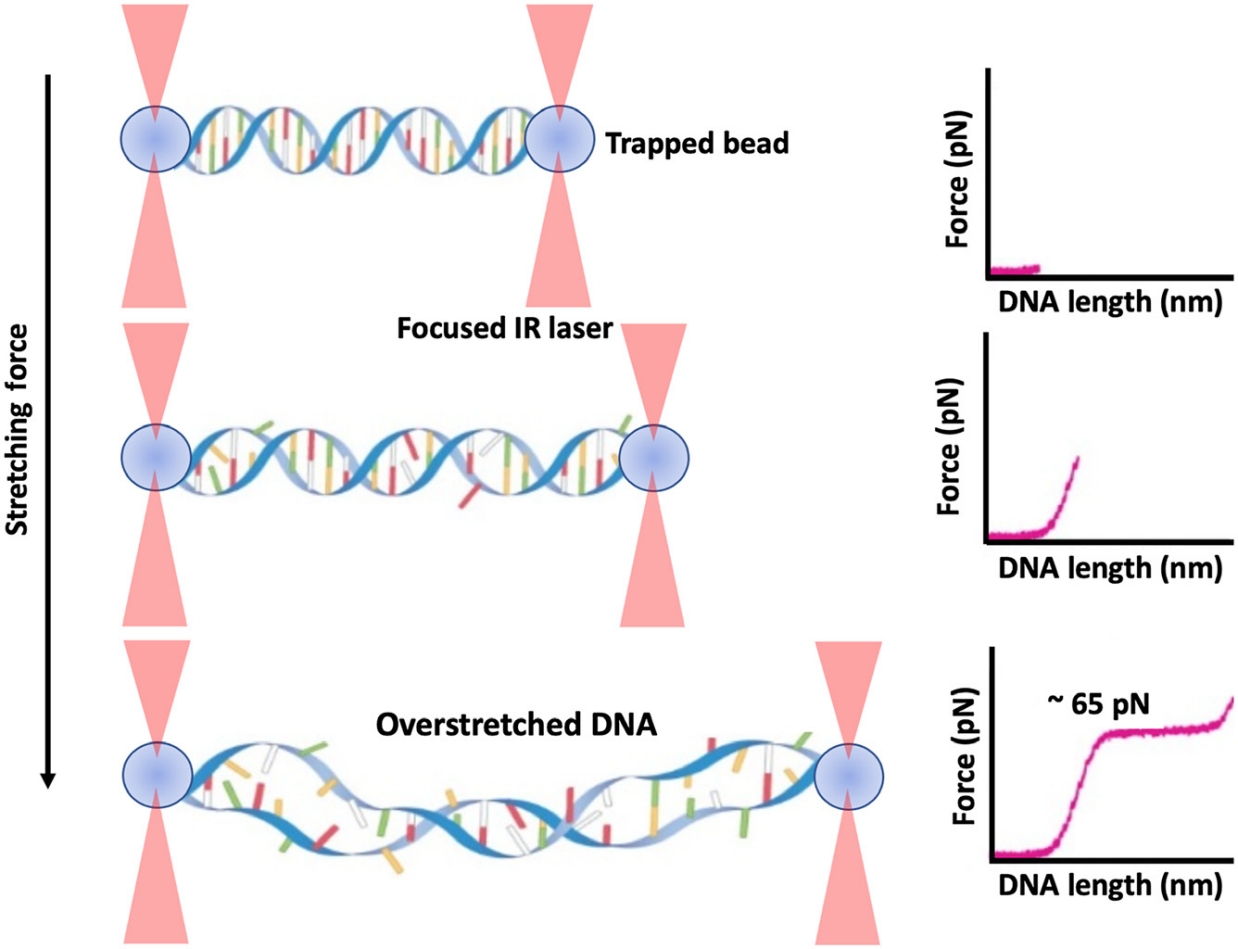
Schematic of a DNA molecule stretched between two optically trapped beads. As the stretching force is increased, the DNA length increases until it reaches the overstretching point (at ∼65  pN).

The resolution to this question was provided, using a dual-beam optical tweezers setup combined with the fluorescence microscopy, in separate studies by Van Mameren et al.^38^ and King et al.^39^ Both studies used an intercalating dsDNA-specific dye and ssDNA-binding proteins as fluorescent markers to record fluorescence images while pulling the ends of a single DNA molecule with optical tweezers. However, Van Mameren et al., differently from King et al., combined optical tweezers and fluorescence microscopy with microfluidics. They used a microfluidic flow chamber containing the dsDNA-specific dye to visualize the double-stranded DNA segments. Both research groups were capable of visualizing the structural changes of the molecule undergoing the overstretching transition. Both torsionally constrained and unconstrained molecules were investigated, confirming that in both cases a force-induced melting of the dsDNA into ssDNA unambiguously occurs in the overstretching transition. A schematic depicting the phenomenon of the overstretching of a DNA molecule attached to two optically trapped beads is shown in Fig. 4.

These results were also confirmed by pulling experiments conducted by Zhang et al.,^40^ which calculated the stiffness of the overstretched DNA applying forces >70  pN using a calorimetric magnetic tweezers setup. The same results were later supported by molecular dynamics simulations.^41^ The kinetics of the overstretching transition and its force-dependence were also investigated with optical tweezers.^42^

Optical tweezers have also been extensively used to study the folding process of proteins.^43^ However, only recently have major advances been made in mapping the free-energy landscape (FEL) of the protein using this technique. The FEL is a map of the free energy across the configuration space of a protein. A protein can theoretically exist in a nearly infinite number of conformations along its energy landscape, but in reality folds only into secondary and tertiary structures to which the minimum free energy is associated. To function correctly, proteins must be correctly folded into specific 3D shapes through the process of protein folding. Understanding the FEL, which underlines the thermodynamics and kinetics of such a process, is therefore essential. However, for completeness, it is important to state that to derive the complete FEL it is necessary to give careful statistical consideration of less-probable events, such as the folding into secondary and tertiary structures.^44^^–^^46^ Interestingly, deconvolution methods coupled to optical tweezers experiments have allowed the reconstruction of complete FEL. Deconvolution is used to remove the compliance effects of the instrument and the DNA handles, determining the point spread functions describing the distribution of measured extensions around the mean which is caused by components other than the protein of interest. The folding energy landscape of the GCN4 leucine zipper protein was mapped using such an approach.^47^ Stigler et al.^44^ exploited optical tweezers to map the complex folding pathways of calmodulin, which is a calcium-modulated protein. They combined the use of highly stable optical tweezers measurements of full-length and truncated calmodulin variants with hidden Markov model (HMM) analysis resolving six distinct states along the folding pathway. For a comprehensive explanation on the HMM, we refer the reader to the tutorial by Rabiner.^48^ The author determined the kinetic connectivity of all the intermediate folding states and the HMM analysis derived lifetimes and thermodynamic stabilities of them. This led to a detailed folding energy landscape of the molecule.

Cecconi et al.^49^ used optical tweezers to study the protein RNase H (ribonuclease H). RNase H is an ubiquitous cellular ribonuclease that recognizes a DNA/RNA duplex and degrades the RNA strand of this duplex. They derived the force-extension curves by stretching and releasing the protein several times by moving a micropipette with respect to the optical trap. The protein transitions from the folded to the unfolded state and vice-versa are associated with a jump in force. They identified the presence of an intermediate state along the folding pathway. They also observed hopping phenomena between the intermediate and native states by carrying out force-clamp experiments. In this way, they derived the kinetic rates of each state from the observed lifetimes defining the FEL of the protein. Later, a similar approach was used by Alemany et al.^45^ to study the folding and unfolding mechanisms of the protein Barnase, which is synthesized by the *Bacillus amyloliquefaciens* bacterium. The authors analyzed the force-induced protein folding using the Bell–Evans (BE) and Dudko–Hummer–Szabo (DHS) models.^50^^–^^56^ Both models assume that the protein needs to overcome a single kinetic barrier in the FEL to unfold. However, the BE model assumes that the position of the transition state relative to the native state does not depend on the force, while assuming that the height of the kinetic barrier does. The DHS model instead considers either a parabolic or a linear-cubic-like shape of the FEL and assumes that the force decreases the energy by certain amount proportional to the extension of the given molecular configuration. The authors assumed that the protein, during its conformational transition, has to overcome a single energetic barrier between the native and the denatured states. The height of this energetic barrier was obtained by mapping the distributions of unfolded forces, which led to the determination of the force-dependent kinetic rates of the protein.^45^

By means of pulling and releasing experiments, Heidarsson et al.^46^ investigated the neuronal calcium sensor-1 (NCS-1), which is a protein associated with cognitive processes and increases in bipolar disorder and some forms of schizophrenia.^57^ The authors determined the kinetics of the various transitions and the FEL of the molecule using both an HMM algorithm and the Bell model.

Besides the remarkable results presented already, optical tweezers have been central to numerous other single-molecule investigations. As an example, major advances have been made in the investigation of RNApol, which is fundamental in the transcription of DNA. In particular, Fazal et al.^58^ used a dual-optical tweezers trap to investigate the initiation stage of DNA transcription by RNA polymerase II. They attached the complex made by the polymerase and the transcription factors to one bead and the DNA template to another bead. The change in distance between the two optically trapped beads was monitored by the authors with subnanometric resolution as soon as the initiation stage started. However, to track the molecular trajectory of the single polymerase molecule over long distances and extended times, a reduction of the noise typically associated with dual-beam optical tweezers is necessary. Recently, Righini et al.^59^ achieved a reduction of such noise using a new approach to investigate the initiation process consisting of a “time-shared” dual-optical trap (where the two traps are formed by a single laser beam whose direction is switched at high rates by an acousto-optic deflector) combined with an HMM algorithm. A similar but more accurate approach is used by Righini et al.^59^ and Gabizon et al.^60^ In their works, a near-infrared laser was passed through an acousto-optic deflector alternating its position between two traps every 5  μs. BFPI was then used to measure the position of the beads relative to the traps. The improvement with respect to previous works lies in the use of a nonparametric computational method for data analysis. Due to the high spatiotemporal resolution of this method, they were capable of investigating the dynamics of pausing events in the elongation stage down to the 100-ms time scale, determining in real time the position of the *E. coli* polymerase on the DNA template.

### 3.2 Near-Field Optical Tweezers for the Trapping and Manipulation Small Biomolecules

The precise direct manipulation and regulation of single molecules as small as a few nanometers is now possible due to the development of the near-field nanotweezers, such as plasmonic or photonic crystal optical tweezers. These enable stable trapping and precise manipulation beyond the diffraction limit of light.^61^^,^^62^ We refer the reader elsewhere for further discussion of the use of materials science and the near-field for trapping.^22^

The traditional optical tweezers make use of a tightly focused propagating laser beam to trap particles near the focal point due to the gradient force. However, the gradient force is limited so makes it generally hard to trap small objects. Therefore, it would be easier if the forces exerted could be increased by creating stronger field gradients. This need has given rise to near-field optical traps, which create the subwavelength focusing required to generate strong gradient forces using fields generated by the plasmonic or dielectric nanostructures.

The plasmonic optical tweezers use the phenomena of surface plasmon polaritons, which use collective charge oscillations at metal–dielectric interfaces or bound electron plasmas within metal nanostructures, to enhance optical confinement and the trapping potential.^63^ However, for a long time, the application of plasmonic optical tweezers has mainly been limited to trapping of metallic or dielectric nanoparticles and hampered due to localized heating and the photothermal effect produced on the plasmonic surface.^64^ Plasmon waves are powerful, but the key problem is their dissipation results in heat. The resultant thermal forces can easily outweigh optical forces. This local heating effect has, therefore, been seen as an obstacle to stable trapping of particles on a plasmonic substrate. Therefore, different strategies to solve this problem have been explored. In particular, the integration of a heat sink to dissipate excess heat^65^ or off-resonance excitation to minimize light absorption^66^ has been used. Moreover, the design of various dynamic plasmonic nanostructures generating less heat has been established.^64^^,^^65^

These advances now mean we can perform a different type of molecular study in an optical trap, not requiring tethering of the molecule to a microparticle. A good example is the direct trapping of single proteins using the double-nanohole structures that have been the focus of several studies.^67^^–^^71^ Pang and Gordon^67^ used a double-nanohole structure, realized with focused-ion beam lithography, to trap and unfold single bovine serum albumin (BSA) molecules with a hydrodynamic radius of only 3.4 nm. A couple of years later, Balushi and Gordon^71^ made a step forward using the double-nanohole plasmonic trapping for a real-time detection of protein–small molecule interactions in a label-free way. Balushi and Gordon subsequently studied the protein–ligand interactions between human serum albumin and tolbutamide, as well as phenytoin.^72^ Very recently, the group developed a new inexpensive colloidal-based nanofabrication approach for the double-nanohole structures.^73^

**Fig. 5:**
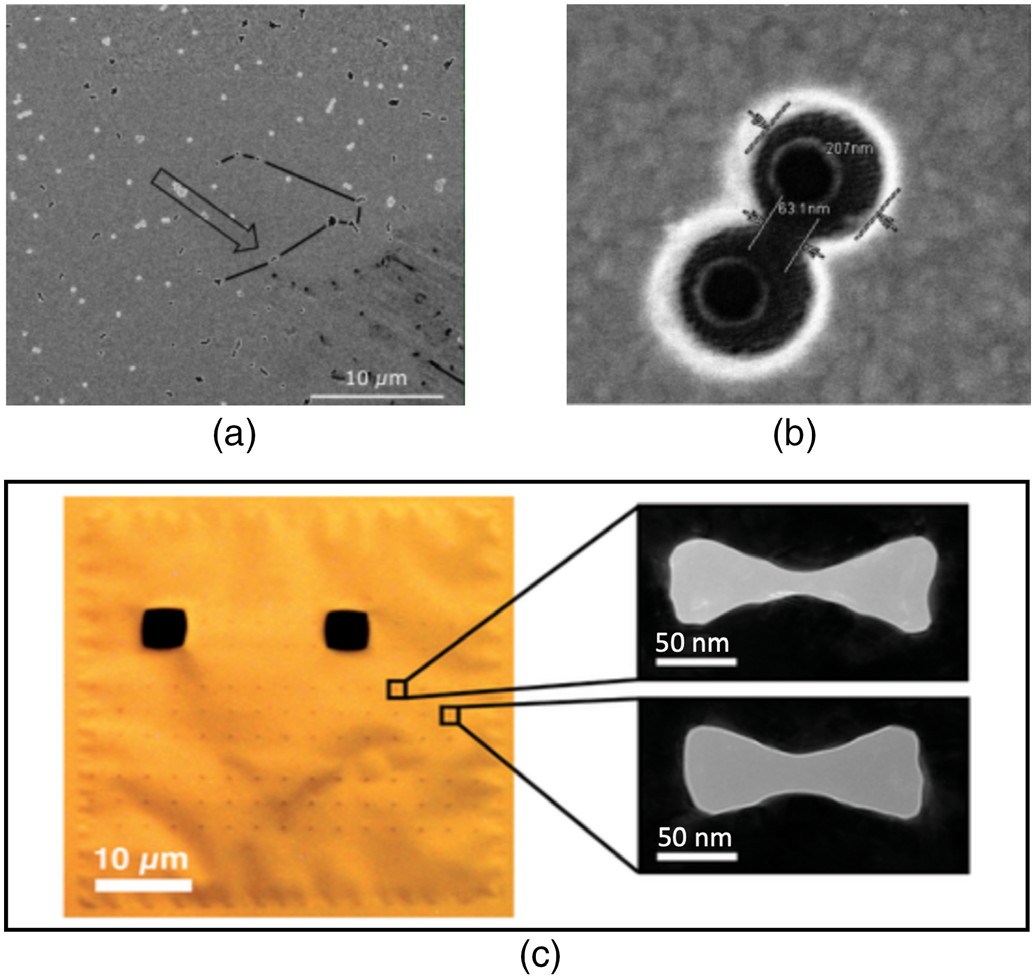
Plasmonic nanostructures used for optical trapping. (a) An SEM image indicating the position of a double-nanohole aperture. (b) Zoom in of a double-nanohole with 60-nm gap size. Adapted with permission from Figs. 3(a) and 3(c) of Ref. 73, © 2019 OSA. (c) Optical image of a plasmonic nanopore membrane. The two black squares are apertures for detector alignment. The faintly visible dots represent the nanoantennas. On the right, two zoomed in TEM images of inverted-bowtie plasmonic nanopores with different geometrical features. Adapted with permission from Figs. 3(a) and 3(c) from Ref. 74, © 2019 Wiley.

With such a structure, the authors trapped single rubisco protein and BSA. Verschueren et al.^74^ increased control over single-molecule trapping by combining the force of optical trapping with an electrophoretic force in a plasmonic nanopore biosensor. They managed to trap individual beta-amylase proteins as well as a 200-kDa enzyme. Figure 5 shows the scanning electron microscopy images of the double-nanohole structure used by Ravindranath et al.^73^ and the plasmonic nanopore membrane used by Verschueren et al.^74^

Different designs of plasmonic tweezers have also been used to immobilize DNA, which is essential for the development of DNA chips. For example, Shoji et al.^75^ developed gold nanopyramidal dimer arrays on glass substrates, whereas Belkin et al.^76^ developed a solid-state nanopore featuring a gold bow tie structure.

Photonic crystal optical tweezers also enable stable trapping at the nanometer size. A photonic crystal resonator (PCR) contains regularly repeating regions of high- and low-dielectric constants (photonic band gaps), which allow or forbid light to propagate in a certain frequency range. When a laser beam at the resonant wavelength enters a PCR, a stationary interference pattern forming a localized and amplified optical field within a subwavelength volume is generated inside the PCR, which leads to an extremely strong field gradient in three dimensions for nano-object trapping. Photonic crystal-based optical tweezers have already shown their potential of trapping biomolecules without damaging them. Furthermore, their ability to confine biomolecules in subwavelength trapping volumes without subjecting them to the temperature increases associated with plasmonic tweezers opens up new perspectives for studying protein–protein and protein–DNA fragments interactions. Photonic crystal structures can also be integrated with microfluidic systems to create lab-on-a-chip platforms for the sorting and/or manipulation of biological molecules and do not have the disadvantage of significant thermal effects as seen with plasmonic based approaches.^77^

**Fig. 6:**
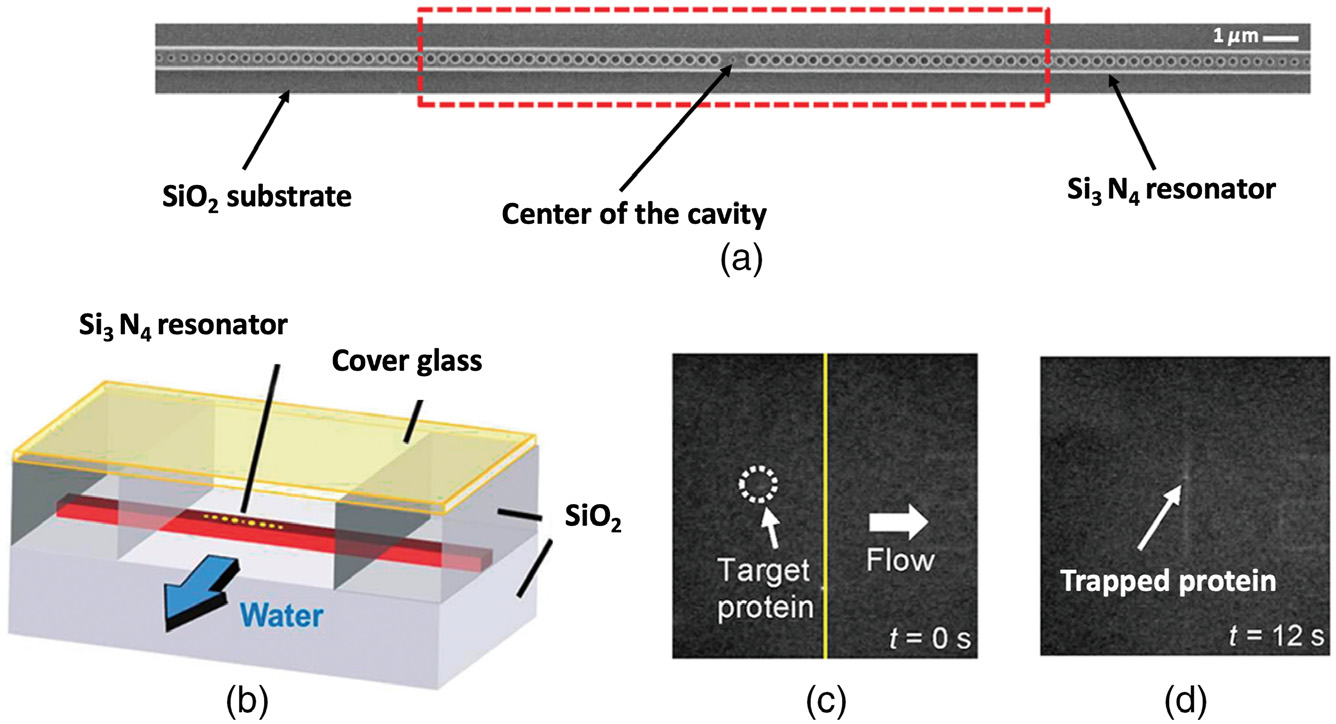
Photonic crystal optical tweezers. (a) Scanning electron microscope image of a silicon nitride PCR. The region in which the electric field enhancement is increased to reach a maximum intensity in the center of the cavity is indicated by the region inside the red dashed lines. (b) Diagram showing the relative locations of the resonator and the flow chamber. (c) and (d) The trapping of a Wilson disease protein on a silicon nitride photonic resonator. The protein (indicated by arrows) is trapped when it arrives at the vicinity of the optically excited resonator. Adapted with permission from Ref. 78, © 2012 American Chemical Society.

Special PCRs capable of trapping nanometer-size objects with minimal temperature rise have been designed by Chen et al. (Fig. 6).^78^ In their work, the authors showed that switching from Si to ${\mathrm{Si}}_{3}N_{4}$ devices and from 1550 to 1064 nm as excitation wavelength greatly reduced the local heating at the PCR given by the optical absorption of water. This new form of a photonic crystal “nanotweezers” allowed the trapping and release of single molecules of Wilson disease proteins in a controlled way in addition to the trapping of streptavidin-coated quantum dots and 22-nm polymer particles. Recently, the use of photonic crystal optical tweezers found their applicability for trapping eukaryotic and bacterial cells.^79^ In this work, the top of a two-dimensional (2D) photonic crystals structure was illuminated with a gently focused 1060-nm laser beam. In this way, the authors were able to create low-intensity optical traps above the surface of the 2D structure to stably capture living cells, maintaining their viability for more than 30 min.

### 3.3 Toward Single Virus Detection

Numerous human diseases are caused by the spread of infectious viruses around the world. They can cause illnesses ranging from the common flu (influenza) to more severe diseases such as the recent novel coronavirus (COVID-19). Since the global pandemic of COVID-19 disease, which has spread very rapidly around the globe, the serious consequences of airborne transmission of viruses have caused great concern. The compelling need for controlling the spread of the current and future epidemics has led to huge efforts in developing rapid and efficient virus screening tools.^80^^,^^81^

The use of optical tweezers for trapping individual viruses dates back to 1987^82^ but their application in this field has only been properly explored in the last decade, particularly using the near-field innovations of the trapping geometry. Pang et al.,^83^ for example, used optically trapped and characterized human immunodeficiency viruses with 154-nm mean diameter in culture fluid, by simultaneous use of two-photon fluorescence imaging, revealing their size heterogeneity. Maruyama et al.^84^ managed to trap and transport single influenza viruses of ∼100  nm in size using optical tweezers in conjunction with dielectrophoretic force on a microfluidic chip. Recently, the optical trapping of polymer beads of different diameters between 100 and 3000 nm on planar (F-Si) and nanostructured (BSi) crystalline silicon was investigated for possible implications in bacteria and virus trapping.^85^ The authors demonstrated that BSi surfaces can enhance the optical gradient force required to trap 100-nm nanoparticles in contrast to F-Si surfaces. While these studies have been carried out with the traditional optical tweezers, the small size of most viruses and the low-RI contrast with the surrounding medium necessitate the use of near-field optical tweezers (as introduced in Sec. 3.2). In fact, very recently optical trapping of a virus particle of 25 nm (PhiX174) has been achieved by Burkhartsmeyer et al.^33^ by focusing the trapping laser onto double-nanohole apertures created on a gold film.

## 4 Advances in Optical Trapping and Manipulation of Cells

Optical trapping has been widely utilized not only for single-molecule studies but also for larger scales traps and manipulates single cells. The increase in precision and sensitivity with which forces can be measured in optical tweezers has led to their use in a variety of biomedical research and clinical applications, including cell transportation, cell reorientation, cell sorting, and characterization of the mechanical properties of cells. In particular, the last decade saw the application of this technique to the manipulation of cells in crowded biological environments and to *in vivo* biological experiments. This is in part due to the use of advanced beam shaping, the combination of optical trapping with advanced imaging techniques, and advances in materials science that have led to the development of more sophisticated particles including birefringent rotating particles. We discuss a few of these applications.

### 4.1 Measuring the Mechanical Properties of Cells

Optical traps have an important role to play in probing the mechanical properties of cells and their substructures.^86^^,^^87^ Their versatility allows manipulation as well as the measurement of forces, which can be applied from the outer plasma membrane through a force probe trapped and bound to it, or inside the cell by trapping intracellular organelles. The most common modalities used to fully characterize the viscoelastic properties of cells using optical traps are nanoindentation, cell stretching, and tether extension. In cell stretching experiments, both dual counterpropagating trap and optical tweezers can be used to globally stretch cells or subcellular structures and study their elastic and viscoelastic properties.^88^ Tether extension is achieved by attaching a trapped microbead to the membrane and applying a force by moving either the stage or the trap.^89^ As the bead is withdrawn at constant velocity, a long nanotube known as a membrane tether is formed and stretched between the cell membrane and the bead. Pulling out membrane tethers yields important information about cell membrane elasticity. The first use of optical tweezers for tether extraction from cell membranes dates back to the experiments carried out by Ashkin and Dziedzic in 1989.^90^ In nanoindentation experiments, the mechanical properties of the cell, defined through a mechanical model, are derived by measuring a cell deformation induced by an external force probe.^89^

While the basic approach for cell stretching has remained the same in such studies, substantial innovation has been introduced in nanoindentation experiments. In particular, various configurations to perform nanoindentation with optical tweezers on cells have emerged. In 2013, Dy et al.^91^ performed indentation axially by moving the trap toward the cell in a linear way. They measured the stiffness of a Balb3T3 cell using the Hertz model. Such a model is the most frequently used to derive the cell’s mechanical properties from measurements of force and indentation depth in optical tweezers experiments. The trap was then moved in an oscillatory way in a later experiment of axial indentation performed by Falleroni et al.^92^ They employed an oscillatory optical trap to apply gentle forces perpendicularly to the cell membrane mechanically stimulating mouse neuroblastoma NG108-15 cells. They demonstrated that such periodical stimulation induces cellular calcium transients that depend on the strength of the stimulus. Axial indentation was also performed by Coceano et al.^93^ and by Yousafzai et al.^94^ The authors in these two separate studies moved the sample through a piezoelectric stage against a trapped dielectric microsphere to perform the indentation.

**Fig. 7:**
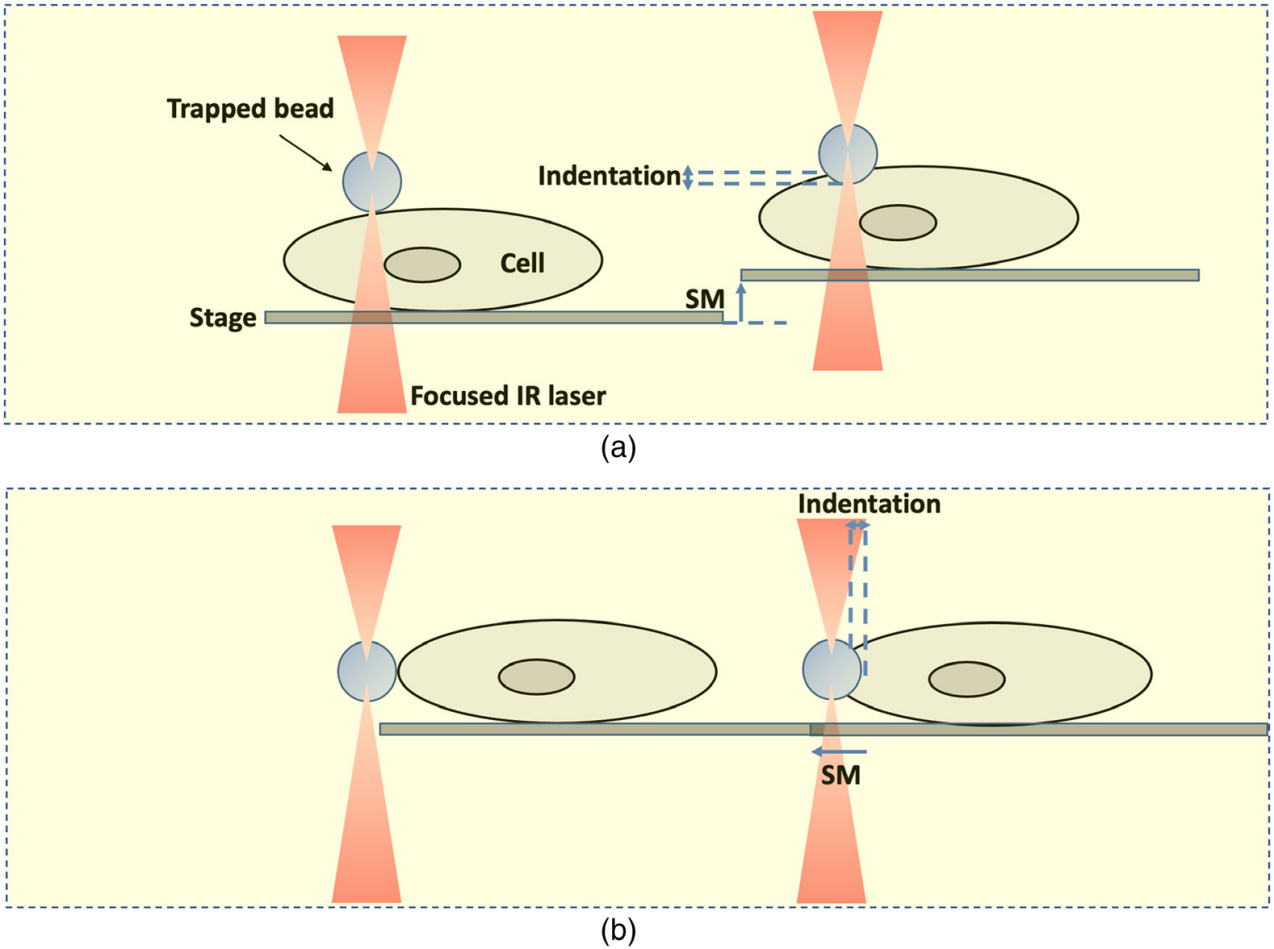
Schematic showing cell indentation by optical tweezers. (a) Axial indentation in which the stage is moved up by the distance SM. (b) Lateral indentation in which the stage is moved laterally by the distance SM. In both configurations, the cell interacts with the bead displacing it, while the bead indents the cell.

Lateral indentation, which implies the movement of the trap along the image plane against a perpendicular cell membrane, has instead been used by Zhou et al.^95^ They measured cell elasticity as an indicator of cellular alterations that can occur in myelogenous leukemia cells. For completeness, it is worth mentioning that axial indentation can be performed also by moving the stage axially against a trapped bead until contact with the cell is reached. The indentation is produced by the resistance of the bead to the cell movement. Figures 7(a) and 7(b) show the different configurations to perform the axial and lateral indentations, respectively.

In recent years, optical tweezers have also played a pivotal role in the investigation of mechanotransduction signals in living cells and organisms. These processes are essential for the cell to adapt to the continuous dynamic modifications of the microenvironment. The intracellular molecular processes, through which mechanical stimuli arising from the surrounding extracellular matrix or from neighboring cells are transformed into biological responses, are collectively named “mechanotransduction.” In optical tweezers experiments, trapped microspheres are coated with adhesion molecules such as fibronectin or cadherin to stimulate mechanical forces of different intensity and frequency, focal adhesions, or adherence junctions on the plasma membrane. Intracellular mechanotransduction pathways in human mesenchymal stem cells (hMSCs) have recently been investigated by Kim et al.^96^ These authors used optical tweezers combined with a genetically encoded FRET-based calcium reporter to investigate force-induced calcium signals at different subcellular locations. They used fibronectin-coated beads to apply a mechanical force of 300 pN to hMSCs inducing ${\mathrm{Ca}}^{2+}$ release from the endoplasmic reticulum both through the cell cytoskeleton and the plasma membrane, revealing that the mechanotransduction at different depths of the cell body is mediated by differential sets of mechanosensing elements. More recently, Feng et al.^97^ trapped pMHC-bound beads with different pMHC surface concentrations in the proximity of T lymphocytes, applying pN forces to investigate chemical thresholds depending on applied load and force direction. A much higher pMHC density than observed physiologically was required to activate T cells receptors (TCRs) in the absence of force. On the contrary, only two pMHC molecules at the interaction surface were involved in their activation when a force of ∼10  pN per TCR molecule was applied in the shear direction. These findings support a mechanosensitive-based regulation of cell activation. They also demonstrate the potential of optical tweezers for mechanotransduction signal investigations.

### 4.2 In Vivo Cell Trapping

The survival and normal functioning of living organisms while and after being trapped and manipulated were demonstrated by Ashkin only a year after the invention of optical tweezers.^82^ After his studies, many other works investigating the localized and potentially harmful heating caused by the absorption of light from trapped molecules and cells were carried out. To date, we know that the use of the near-infrared (IR) region, with wavelengths between 700 and 1300 nm, the absorption and subsequent heating (damage) of biological samples is minimized.^98^^,^^99^ Therefore, most biological optical trapping experiments are conducted using lasers in this region.

In the last decade, optical tweezers have emerged as a useful tool for the particular case of trapping and manipulating living cells *in vivo* to gain additional physiological insights at the single-cell level inside complex multicellular organisms. This requires judicious choice of sample and conditions given the very weak nature of optical forces. The trapping and manipulation of cells within living animals have been achieved by Zhong et al.^100^ They trapped and manipulated flowing red blood cells in the blood capillaries of living mice. They also managed using optical tweezers to clear the blockage of a capillary (due to the presence of red blood cells amalgamating together), thus recovering the blood flow. Later, Johansen et al.^101^ trapped and manipulated fullscale nano- to micron-sized structures inside a living zebrafish embryo. They demonstrated for the first time active micromanipulation of injected nanoparticles for nanomedicine applications and injected bacteria to stimulate the immune response. They also trapped and manipulated naturally occurring zebrafish cells such as erythrocytes and macrophages.

**Fig. 8:**
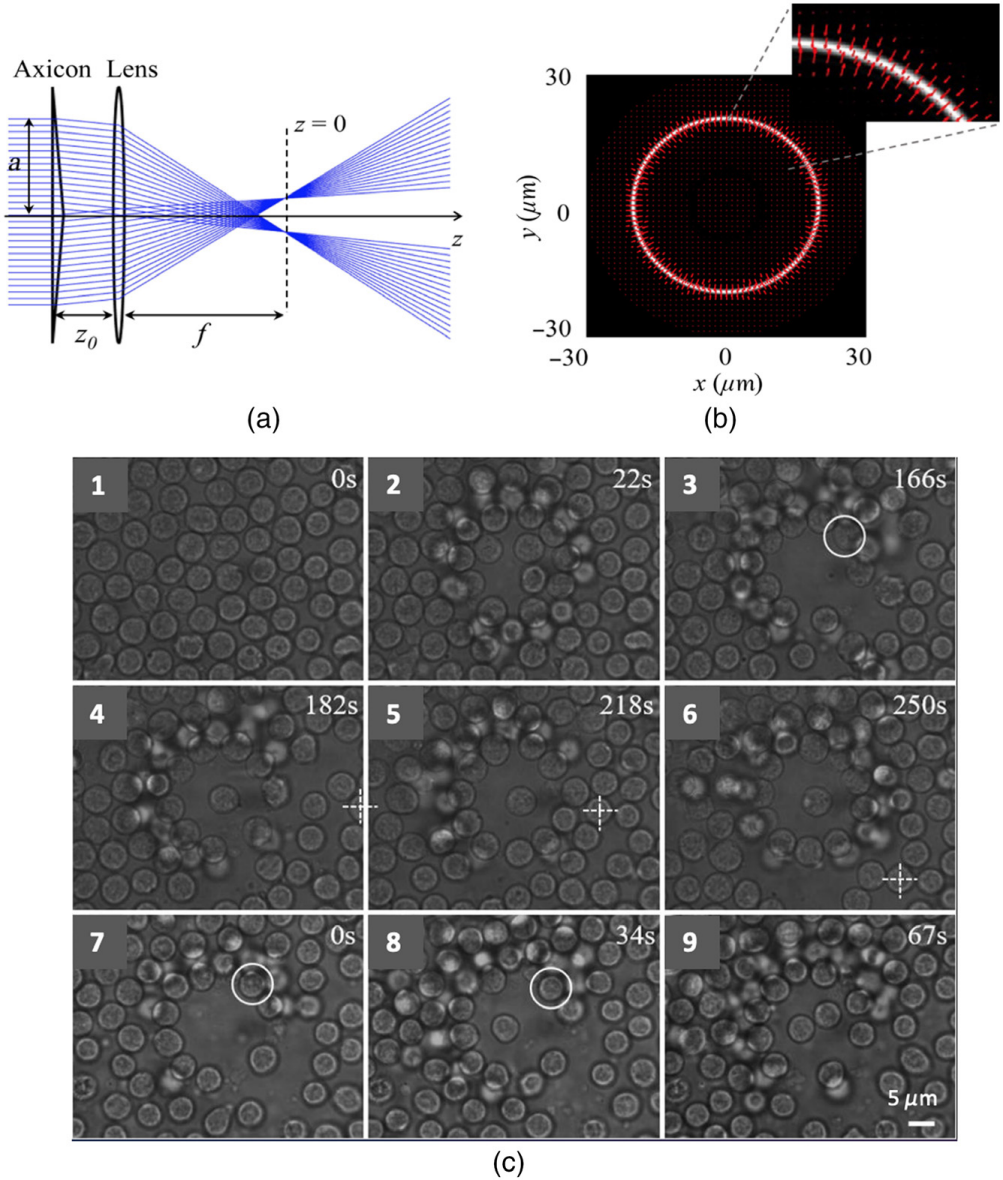
Optical trapping in crowded environments. (a) Experimental scheme for an adjustable optical shield. The hollow beam can be produced behind the lens when $z_{0}<f<Z_{\max}$, where $z_{0}$ is the distance between axicon and lens, f is the focal length of the lens. (b) Force distribution of optical shield. The annular intensity distribution at the focal plane is calculated as a function of a particle’s position over x−y plane using ray optics model. The particle has a radius of 2.5  μm and RI of 1.50 and is immersed in water. The red arrows show the magnitude and direction of total force vectors as a function of the position. (c) Manipulation of live lymphocytes ejected from a lymph node. (1) A dense distribution of lymphocytes. (2), (3) A blank area is gradually created with the annular beam. (3), (4) The cell in the white circle is trapped and moved to the center. (4), (5) The trapped cell is moved to the reference point indicated by a cross. (7)–(9) Independent manipulation of two lymphocytes in a crowd of lymphocytes. Adapted from Figs. 1 and 4 of Ref. 102 (CC BY 4.0).

However, one of the main challenges of trapping cells *in vivo* remains the crowded environment inside living animals. In this condition, in fact, while trying to trap and manipulate individual cells *in situ*, various classes of cells ambiently present frequently enter the trap, thereby reducing the accuracy of the measurements. To overcome this limitation, different strategies have been exploited over time, most notably the use of beam shaping to generate propagation invariant beams that exhibit “self-healing properties” while overcoming the limited depth of focus of Gaussian beams. To avoid the disturbance from crowded environments during individual lymphocytes manipulation inside a lymph node, Zhao et al.^102^ used an annular beam serving as an optical shield. The structured light beam was created using an axicon to produce a Bessel-like beam that was then focused using a converging lens to create an annular beam as shown in Fig. 8(a). The optical shield was regulated by adjusting the distance between the axicon and the lens. The hollow beam can clear a blank area, at the center of which a trapped individual cell can be shielded. When the hollow beam is switched on, cells around the beam center are subjected to a drag force and pushed outward to the high-intensity ring where the transversal trapping efficiency and longitudinal trapping efficiency are balanced. The force distribution of the optical shield is shown in Fig. 8(b). After such an optical shield is established, an additional optical trap is used to capture and manipulate a single cell in the center. As an example, the manipulation of live lymphocytes ejected from a lymph node is shown in Fig. 8(c).

### 4.3 Use of Rotating Particles to Manipulate and Study Cells

In recent years, light fields with spin angular momentum have been used in optical trapping to rotate birefringent particles in addition to translating them.^103^ Birefringent particles are optically anisotropic objects having an RI that depends on the polarization and particular propagation direction of the light. Circularly polarized light carries spin angular momentum that can be transferred to those particles. During passage of such a beam through a birefringent particle, the polarization state changes resulting in an optical torque being applied to the particle which is then set into rotation. A terminal angular velocity is reached, dependent upon the rotational Stokes drag of the immersion liquid. In the last decade, the use of rotating particles has made advances in biology, opening up the opportunity for new studies, including those in microfluidics and neuroscience.

In microfluidic chips, micron-size birefringent particles are often used as the optical rotation probes for local rheology measurements.^104^ These rotate due to an optical torque resulting from a change in polarization from the trapping beam. Torque measurements have been used for the measurement of forces or viscoelasticity properties of fluids and biological systems.^105^^–^^107^ The total torque on a spherical particle can be precisely measured by knowing the viscosity surrounding a particle and the initial angular momentum imparted to the particle itself. However, if the relationship between cell’s behavior and their host environment needs to be investigated, smaller nanoscale probes of viscosity are required. Nonetheless, as the size of particles made of birefringent material reduces below the submicron scale, the transfer of optical torque becomes negligible and insufficient to induce their rotation.^108^ Therefore, lately, major efforts have been made on developing nanoparticles of different and more complex compositions. Recently, the use of eccentric submicron scale core-shell particles has been numerically investigated demonstrating their potential of rotating with moderate light powers not achievable with currently used birefringent particles. The authors show that breaking centrosymmetry in core-shell particles leads to increased optical torques.^109^

In the area of neuroscience, birefringent rotating particles have been successfully used as motors to guide neuronal growth. The behavior of the growth cone at the tip of the growing axon controlling the direction of axonal growth and migration has been investigated by Wu et al.^110^ They created a localized microfluidic flow and thus a shear force using birefringent spheres trapped and set into rotation in close proximity to the growth cone. The cone’s development was influenced by the rotation direction and location of the trapped sphere. In this way, they have shown the influence of environmental dynamics on neuronal growth.

### 4.4 Optical Trapping Combined with Imaging

Optical tweezers are highly compatible to be incorporated with various imaging approaches including, but not limited to, brightfield, phase-contrast, and fluorescence microscopy. Ongoing advances in optical trapping, as well as parallel improvements in imaging and adaptive optics, have significantly broadened the capabilities of optical tweezers, thereby increasing their use in biological studies.^61^ In particular, the past decade has seen optical tweezers becoming an established tool in neuroscience, opening up the opportunity to provide further insight on molecular and neuronal dynamics, as well as on the functioning of the neural activity of model organisms as a whole.^111^

**Fig. 9:**
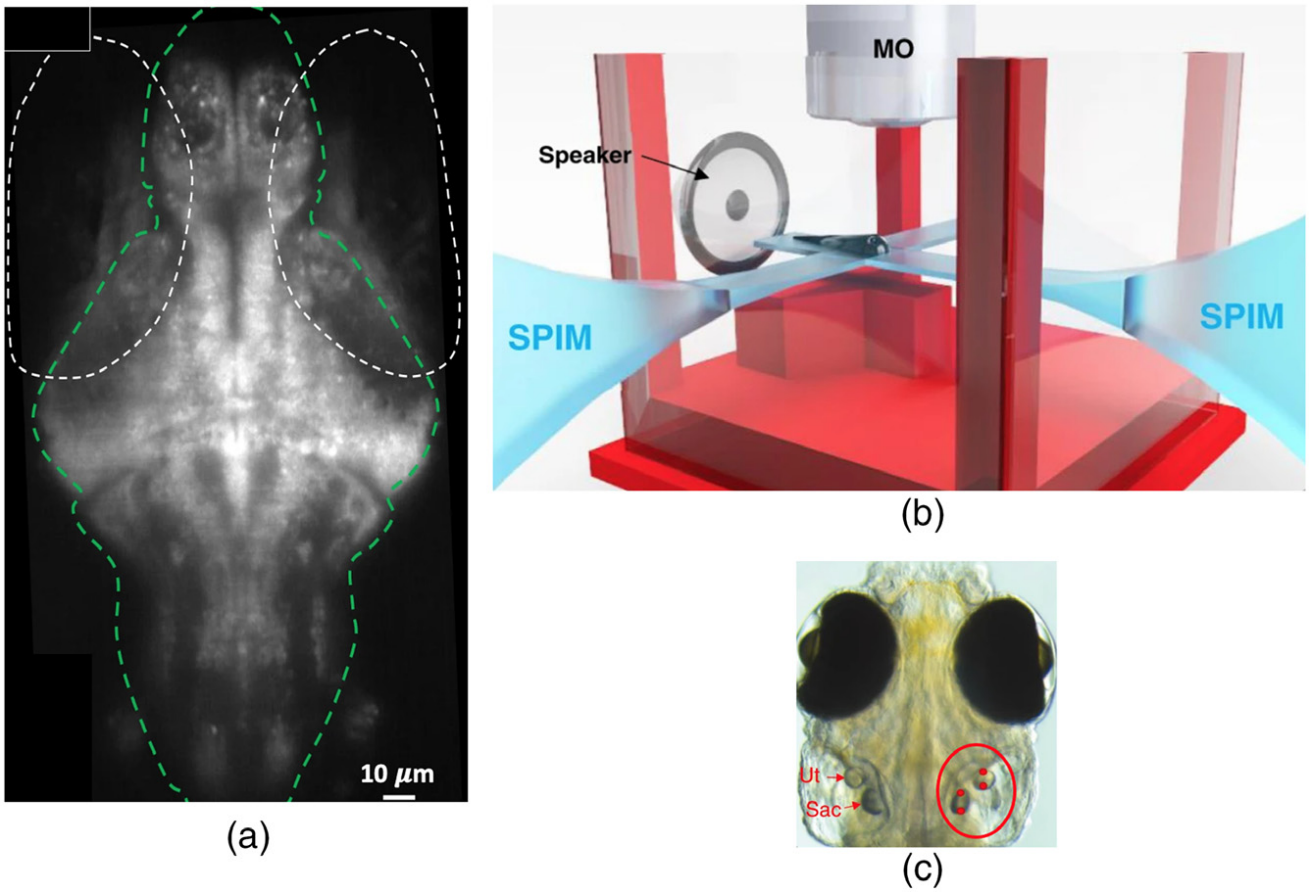
Optical manipulation of otoliths in larval zebrafishes. (a) Fluorescence image recorded from one plane in a zebrafish. The white dashed ovals indicate the eyes, and the green line delineates the brain. (b) Schematic showing the position of a larva in the setup with the SPIM planes, the trapping microscope objective (MO), and the location of the speaker. (c) Location of the optical traps (red dots in the red circle, right) within the utricular (Ut) and saccular (Sac) otoliths. Adapted from Figs. 1(b)–1(d) of Ref. 113 (CC BY 4.0).

Trapping with large-scale imaging such as light sheet microscopy is coming to the fore. Bambardekar et al.^112^ exerted optical forces on cell contacts within the early Drosophila embryo. The shape changes led to a direct measurement of forces present at cell contacts and to determine the time-dependent viscoelastic properties of the tissue sample.^112^ Recently, by combining optical trapping with bright field imaging and selective planar illumination microscopy (SPIM), forces have been applied to zebrafish otoliths, which are ear-stones located in the inner ear.^113^^–^^116^ In these studies, the behavior and brain activity was observed in response to the optical manipulation of individual or multiple otoliths in 6-day-old zebrafish embryos. In particular, Favre-Bulle et al.^114^ combined optical trapping of the utricular otoliths with whole-brain calcium imaging for stimulating the vestibular system while mapping the responses in larval zebrafish. The researchers built a custom SPIM setup for volumetric imaging consisting of two scanning light sheets, a fluorescence emission channel, a camera for behavioral imaging, and two 1064-nm beams (one for each otolith) for optical trapping. They generated scanning light sheets using galvanometric control of mirrors and detected fluorescent emissions through the same objective that delivered the optical tweezers beam. In this way, the vestibular system was stimulated, and calcium imaging recorded without the need to move the animal. Each of the otoliths could be stimulated individually or in combination. In a later work, the same group modified their optical trapping setup to allow higher frequency manipulations (10 Hz to 1 kHz) to produce bio-opto-acoustic stimuli.^113^ Vibrations generated by a rapidly oscillated optical trap in individual otoliths were used as a perception of sound, while adjacent otoliths were either left unstimulated or similarly stimulated with a second optical laser trap. Using this technique, the authors of the work demonstrated that all of the four ear-stones can be displaced at a chosen frequency, stimulating the response of neurons to natural tones. A fluorescence image of a single plane in a zebrafish recorded with the light sheet setup can be observed in Fig. 9(a). In Fig. 9(b), a schematic showing the position of the larva in the setup used by the authors is depicted. Figure 9(c) reveals the position of the optical traps within the otoliths.

These studies have shown the potential of the integration of optical tweezers with imaging technologies for behavioral studies in large model organisms.

The simultaneous trapping and imaging in the axial plane is one of the main advances made since the turn of the century in the field of optical trapping combined with advanced imaging. This has potential implications in several research fields including tomographic phase microscopy (TPM) and super-resolution (SR) microscopy,^117^ which are very useful in the study of biological samples. TPM is a label-free technique to measure the 3D RI of an object from which morphological and biochemical information, and from this the dynamics of biological samples, can be extrapolated. The combination of optical trapping and TPM is particularly appealing for studying the morphology and dynamics of living cells in an aqueous solution in a contact-free manner. However, one of the main limitations in using the same objective for trapping and phase imaging is the unstable rotation of the sample while acquiring the phase distribution at various angles to reconstruct the 3D RI. The problem can be solved by rotating the sample around the z axis while imaging it in the axial plane during its rotation, thereby reconstructing the 3D RI distribution with isotropic resolution. SR microscopy is a technique capable of overcoming the optical diffraction limit permitting imaging with a spatial resolution of a few nanometers. Combining optical trapping with SR microscopy allows the immobilization and orientation of individual floating cells which may therefore be imaged from multiple perspectives, thereby permitting the recording of 3D super-resolved information of the sample. The capability of simultaneously trapping and imaging in the axial plane would provide more perspective for investigating the specimens because imaging is not restricted to the x-y plane as in the case in which the same objective is used for both trapping and imaging.

As optical tweezers have matured as a technology, commercial systems have become increasingly available, often with an integrated imaging capability. Examples suitable for lab-based studies range from modular systems with cage-mounted optics,^118^ add-ons for existing microscopes^119^ to complete systems build around a microscope body.^120^ Applications outside the laboratory are also enabled by a range of portable optical tweezers systems.^121^

## 5 Conclusions

The study of single molecules is an area that has seen the successful application of optical manipulation for a long time. The use of optical tweezers has in fact led to major advances in unveiling the structure of the overstretched DNA molecule as well as understanding the thermodynamics and kinetics of the protein-folding process and investigating the dynamics of molecular motors. Future directions may see an increase in the development and use of near-field traps to manipulate single viruses. In the future, there is likely to be a desire for nanostructures capable of trapping larger size particles or a wider dynamic range of sizes. Multiplexing such studies and gaining higher throughput may offer advantages.

An exciting theme to emerge is the use of optical tweezers for *in vivo* experiments to investigate live cell dynamics in living animals and in crowded environments such as in the blood flow.^102^ This is mainly rendered possible by the careful choice of experimental scenario, the development of appropriate optical force measurement techniques, and the use of beam shaping. In the future, this may be further assisted with the use of wavefront correction or fiber-based methods to allow further studies of tweezers inside living organisms.

Combining optical forces with imaging as well as other technologies for exerting forces remains of interest. As an example, the combination of optical and acoustic trapping for the development of multimode or hybrid devices may lead to more versatile manipulation systems capable of acting on different length scales.

As we look forward, it certainly seems that optical forces are being exploited in very powerful ways in the biomedical arena. Though weak, they have been at the heart of some exceptional new biological breakthroughs and more is sure to come as the technology for optical trapping continues to advance.
